# Predictive Value of Soluble Growth Stimulator Gene 2 Protein for Coronary Slow Flow/No-Reflow in ST-Elevation Myocardial Infarction Patients Receiving Percutaneous Coronary Intervention

**DOI:** 10.1155/2022/9322460

**Published:** 2022-04-15

**Authors:** Shu-min Chang, Yan-tan Yu, Bo Luan, Ai-jie Hou, Yong Wang

**Affiliations:** Department of Cardiology, The People's Hospital of China Medical University, The People's Hospital of Liaoning Province, No. 33 Wenyi Road, Shenhe, Shenyang, Liaoning 110016, China

## Abstract

**Background:**

Soluble growth stimulator gene 2 protein (sST2) is associated with heart failure and myocardial infarction; however, the predictive value of plasma sST2 level for coronary slow flow/no-reflow (CSF/NRF) is unclear. This study aimed to explore the predictive value of plasma sST2 levels for CSF/NRF in patients with ST-elevation myocardial infarction (STEMI) who underwent emergency percutaneous coronary intervention (PCI).

**Methods:**

A total of 242 STEMI patients who underwent emergency PCI at our hospital between November 2020 and July 2021 were enrolled in this study. According to the postprocedural procedure, these patients were divided into the CSF/NRF and control groups. Clinical data were collected from both groups and were used to explore the predictive value of serum sST2 levels for CSF/NRF.

**Results:**

Of the total 242 patients, CSF/NRF was observed in 50 patients (20.7%). Statistically significant differences (*P* < 0.05) were observed in age, diabetes mellitus, sST2 level, neutrophil-to-lymphocyte ratio (NLR), fasting blood sugar, preprocedural blood pressure, intraprocedural hypotension, N-terminal pro-B-type natriuretic peptide, MB isoenzyme of creatine kinase (CK-MB), and cardiac troponin I (cTNI). Multivariate analysis showed that the sST2 level, NLR, and intraoperative hypotension were independent risk factors for CSF/NRF. ROC curve analysis showed that the sensitivity and specificity of the sST2 level for predicting CSF/NRF were 68.0% and 75.5%, respectively, when the sST2 level was more than 64.6 ng/mL (AUC = 0.780, 95% CI: 1.003–1.020, *P*=0.009).

**Conclusion:**

For STEMI patients, preprocedural sST2 levels significantly correlated with CSF/NRF occurring in PCI. sST2 level is a potential predictor for CSF/NRF occurrence.

## 1. Introduction

Acute ST-elevation myocardial infarction (STEMI) is a cardiovascular disease with high lethality, and a mortality rate as high as 4–21% has been reported [1]. Percutaneous coronary intervention (PCI) is the main method used to open culprit arteries. However, coronary slow flow/no-reflow (CSF/NRF) may still occur with optimized PCI treatment, which may counteract the clinical benefit of PCI [2]. CSF/NRF has been confirmed to be related to heart failure, arrhythmia, and long-term mortality [3]. Soluble growth stimulator gene 2 protein (sST2) is secreted by cardiomyocytes and fibroblasts under mechanical stress stimulation and/or cardiovascular stress [4]. A higher level of sST2 is associated with a poor prognosis of myocardial infarction. In addition, the sST2 level is used to guide symptom grade, evaluate prognosis, and drug usage for heart failure [5, 6]. However, the preprocedural predictive value of sST2 levels for CSF/NRF has not been reported. This study aimed to explore the predictive value of serum sST2 levels for CSF/NRF in STEMI patients who underwent emergency PCI, providing a basis for preventing CSF/NRF occurrence.

### 1.1. Subjects and Methods

The study was approved by the Institutional Review Board of the People's Hospital of Liaoning Province and complied with the Declaration of Helsinki. All patients signed informed consent before participation.

#### 1.1.1. Subjects

**Figure 1:**
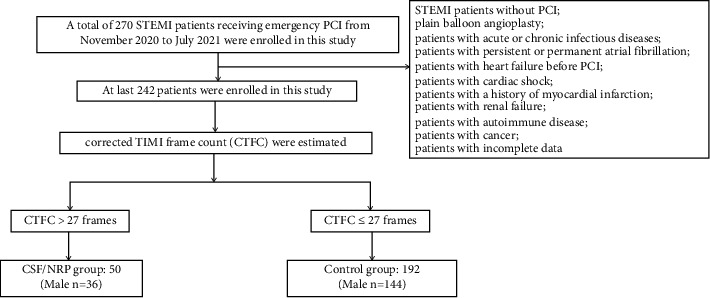
Study flow diagram.

STEMI was diagnosed according to the 2017 European Society of Cardiology guidelines [7]. The exclusion criteria include STEMI patients without PCI, STEMI patients with plain balloon angioplasty, patients with acute or chronic infectious diseases, patients with persistent or permanent atrial fibrillation, patients with heart failure before PCI, patients with cardiac shock, patients with a history of myocardial infarction, patients with renal failure, patients with autoimmune disease, patients with cancer, and patients with incomplete data. Of the total 270 patients with STEMI who underwent emergency PCI in our hospital between November 2020 and July 2021, 242 patients who met the inclusion and exclusion criteria were enrolled in this study (Figure 1). Blood was collected to determine the sST2 level after successful puncture of the radial or femoral artery.

## 2. Methods and Grouping

### 2.1. Coronary Angiography and Coronary Blood Flow Frame Count

All patients were administered aspirin (300 mg), clopidogrel (300 mg), or ticagrelor (180 mg) before PCI, and unfractionated heparin was administered during PCI. The operators determined the periprocedural medication and procedural strategies. The procedures were performed through the radial or femoral artery. The coronary flow velocity was determined using the TIMI frame count method (30 frames/s). It was defined as the first frame when the contrast agent touched the two medial walls of the coronary artery and the diameter of the stained vessel was more than 70%, and it was defined as the last frame when the contrast agent reached the end of each branch of the coronary artery [8]. CSF/NRF was defined as a corrected TIMI frame count (cTFC) of >27 frames for the target vessel during PCI, and patients with coronary artery dissection [9] were excluded [10]. Patients with CSF/NRF were included in the CSF/NRF group and other patients (cTFC ≤27 frames) in the control group. Baseline data such as age, sex, body mass index, smoking history, hypertension history, biochemical indicators, and PCI-related data were collected.

### 2.2. Determination of sST2 Level

In all patients, 2 ml of arterial blood was collected after successful puncture of the radial or femoral artery, stored at 4–8°C, and was used to determine sST2 levels by fluorescent immunoassay and chromatography with a dry immunity analyzer (A2000, Emmy Medical Technology Co., Ltd., Guangxi, China).

### 2.3. Statistical Analysis

Statistical analyses were performed using the SPSS 26.0 software. The measurement data with normal distribution were expressed as mean ± standard deviation (*x* ± *s*), and comparison between the two groups was made using the *t*-test. The measurement data with abnormal distribution were expressed as P50 (P25–P75), and interquartile range and comparison between the two groups were made using the nonparametric Mann–Whitney *U* test. The enumeration data were expressed as percentages, and number of cases and comparison between the two groups was made using *χ*^2^ test or Fisher's exact test. Ranked data were analyzed using the nonparametric rank-sum test. Multivariate logistic regression was used to identify the independent risk factors for CSF/NRF. A receiver operating characteristic (ROC) curve was drawn, and the area under the curve (AUC) was used to evaluate the predictive value of sST2 levels for CSF/NRF occurrence. Statistical significance was set at *P* < 0.05.

## 3. Results

### 3.1. Comparison of Clinical Data between the Two Groups

Comparison of general and laboratory data: of the total 242 patients enrolled in this study, 50 (20.7%) were included in the CSF/NRF group and 192 (79.3%) in the control group. The general and laboratory data of the two groups are given in Table 1. Statistically significant differences (*P* < 0.05) were observed in age, diabetes mellitus, sST2 level, neutrophil-to-lymphocyte ratio (NLR), fasting blood sugar, N-terminal pro-B-type natriuretic peptide (NT-proBNP), MB isoenzyme of creatine kinase (CK-MB), and cardiac troponin I (cTNI). However, the two groups did not differ in sex, smoking, body mass index (BMI), preinfarction angina pectoris, Killip grade, lipoprotein *α* (Lpa), blood uric acid, creatinine clearance(CCr), left ventricular end-diastolic volume (LVEDV), left ventricular end-systolic volume (LVESV), and ejection fraction (EF) (*P* > 0.05) (Table 2).

### 3.2. Comparison of PCI-Related Factors between the Two Groups

Compared with the control group, preoperative systolic and diastolic blood pressures significantly decreased, but intraoperative hypotension significantly increased in the CSF/NRF group (*P* < 0.05). However, the two groups did not differ in the position of myocardial infarction, infarcted arteries, left main coronary artery involvement, diffuse long coronary lesions, Gensini score, stent length, contrast agent dosage, or operation duration (*P* > 0.05) (Table 2).

### 3.3. Independent Risk Factors of CSF/NRF Analyzed by Logistic Regression Analysis

All the factors, including age, diabetes mellitus, sST2 level, NT-proBNP, CK-MB, cTNI, NLR, fasting blood sugar, preoperative blood pressure, and intraoperative hypotension, showing a statistically significant difference between the two groups underwent univariate regression analysis, and the results revealed that sST2 level, CK-MB, cTNI, NLR, SBP, DBP, and intraoperative hypotension were related to CSF/NRF. The above seven factors further underwent multivariate logistic regression analysis, and the results indicated that intraoperative hypotension, sST2 level, and NLR were the independent risk factors for CSF/NRF (*P* < 0.05) (Table 3).

### 3.4. ROC Curves of sST2 Level and NLR for Predicting CSF/NRF

**Figure 2:**
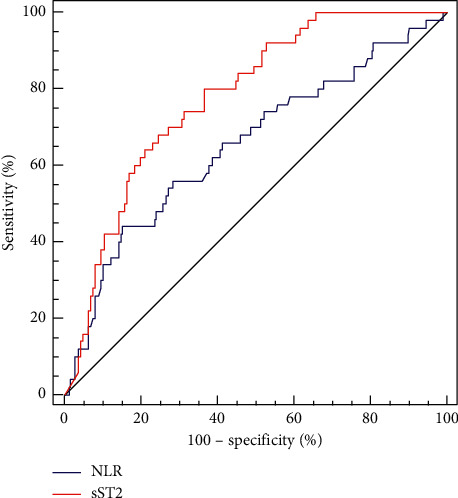
ROC curve showing the distinguishing ability of sST2 and NLR level for CSF/NRF.

ROC curves were used to explore the predictive values of the continuous measurement variables, sST2 and NLR, for the occurrence of CSF/NRF in STEMI patients receiving emergency PCI. ROC curves showed that the sensitivity and specificity of sST2 were 68.0% and 75.5%, respectively, when the sST2 level was more than 64.6 ng/mL (AUC = 0.780, 95% CI: 1.003–1.020, *P*=0.009), and the sensitivity and specificity of NLR were 44.0% and 84.9%, respectively (AUC = 0.654, 95% CI: 1.016–1.158, *P*=0.015). sST2 and NLR can predict values for CSF/NRF, and the predictive value of sST2 was better than that of NLR (Figure 2).

## 4. Discussion

This study indicated that the incidence of CSF/NRF in STEMI patients receiving PCI was 20.7%, which is similar to 12%–32.8% reported by Alidoosti et al. [11]. sST2 and NLR levels have high predictive values for CSF/NRF, especially sST2 level. The sST2 level is easily available; therefore, it may be used to predict CSF/NRF for STEMI patients before PCI.

For STEMI patients, early opening of the culprit arteries can improve patient symptoms and reduce mortality. Although PCI can successfully solve coronary mechanical stenosis in 95% of patients, stenotic coronary arteries fail to achieve effective perfusion owing to CSF/NRF in approximately 5% of patients after PCI [12]. However, the mechanism of CSF/NRF remains unclear. It has been reported that CSF/NRF is associated with tissue edema, free radical formation, neutrophil aggregation, ischemic reperfusion injury, coronary interventional therapy-related vasospasm, and microthrombosis [9, 13]. CSF/NRF increases the risk of postprocedural adverse cardiovascular events. Therefore, effective preprocedural prediction and intraprocedural treatment for CSF/NRF are of vital importance in clinical practice. It has been reported that NLR, Cys-C, and CHA2DS2-VASc scores can predict CSF/NRF occurrence [3, 8, 14]. According to this study, compared with patients in the control group, patients in the CSF/NRF group were older and had a higher proportion of diabetes, higher fasting glucose levels, and lower preoperative blood pressure.

Inflammatory reactions and oxidative stress are involved in various stages of atherosclerosis, are related to endothelial and microvascular coronary dysfunction, and are also associated with CSF/NRF [15, 16]. Interleukin 6 (IL-6) and high sensitivity C-reactive protein (hsCRP) levels significantly increase in both peripheral venous blood and coronary blood of patients with CSF/NRF [17]. Balta et al. [18] found that elevated monocyte levels were associated with CSF/NRF occurrence in patients with STEMI. A recent study showed that the systemic immune-inflammation index predicts NRF after primary PCI [19]. This study indicated that a high NLR level had a predictive value for CSF/NRF occurrence, which was similar to the finding that NLR was an independent predictor of CSF/NRF in COVID-19 patients [14]. The high level of NLR under the action of endothelin-1 increases the ability to adhere to the vascular endothelium, which increases the release of elastase and causes microvascular damage and edema, leading to the occurrence of CSF/NRF.

Similar to a previous study [20], this study indicated that sST2 is an independent risk factor and has a good predictive value for CSF/NRF in STEMI patients receiving emergency PCI. sST2, one of the ST2 isoforms, is released by cardiomyocytes and myocardial fibroblasts under myocardial dysfunction and mechanical stress [21]. When acute myocardial infarction occurs, systolic and diastolic functions in the myocardial necrosis site and ischemic penumbra decrease, and serum sST2 levels markedly increase. sST2 is an inflammatory factor that may activate IL-6 [17]. IL-6 allows inflammatory and endothelial cells to produce tumor necrosis factor alpha (TNF-*α*) and hsCRP, increasing reactive oxygen species in the vascular endothelium. These oxygen radicals can lead to oxidative stress and vascular endothelial damage. The injured endothelium also promotes TNF-*α* and hsCRP release [22], resulting in vicious cycles of oxidative stress, inflammatory response, and CSF/NRF occurrence. It was reported that sST2 levels markedly increased in patients with pneumonia or chronic obstructive lung disease and that sST2 plays an important role in macrophage-mediated inflammatory responses [23]. Neutrophils and macrophages may cause inflammatory reactions and lead to CSF/NRF in STEMI patients.

This study also indicated that intraprocedural hypotension was an independent risk factor for CSF/NRF occurrence in patients with STEMI. Hypotension is associated with ischemia-reperfusion injury. Appropriate perfusion pressure provides the basis for reperfusion. Intraprocedural hypotension changes microvascular blood rheology, and P-selectin and cytokines promote platelet thrombosis, leading to the occurrence of CSF/NRF [24].

This study had some limitations. First, the sST2 levels were measured once. Second, some specific inflammatory factors such as CRP and TNFs were not considered in this study. Finally, it needs to be confirmed with a larger sample size and multicenter studies.

## 5. Conclusion

For STEMI patients, preprocedural sST2 levels significantly correlated with CSF/NRF occurring in PCI. The sST2 level is a potential predictor for CSF/NRF occurrence.

## Figures and Tables

**Table 1:** Baseline characteristics of study population, mean ± SD or *n* (%).

Variable	Control group (*n* = 192)	CSF/NRF(*n* = 50)	*P*
Age (years)	61.2 ± 12.2	66.6 ± 12.0	0.006
Male (*n*, %)	144 (75.0)	36 (72.0)	0.655
Hypertension (*n*, %)	100 (52.1)	20 (40)	0.128
Diabetes mellitus (*n*, %)	47 (24.5)	21 (42.0)	0.014
Smoking (*n*, %)	115 (60.0)	26 (52.0)	0.313
BMI (kg/㎡)	24.5 (22.4, 26.8)	24.7 (20.7, 27.8)	0.774
Preinfarction angina (*n*, %)	31 (16.1)	10 (20.0)	0.518
Killip grade (*n*, %)	—	—	0.578
Killip I	163 (84.7)	44 (88.4)	—
Killip ≥ II	29 (15.3)	6 (11.6)	—
Time to reperfusion (min)	344.0 (196.0, 545.8)	305.5 (208.8, 609.3)	0.967
sST2 (ng/ml)	46.5 (35.6, 64.0)	80.6 (55.1, 121.4)	≤0.001
Peak NT-proBNP (ng/L)	1595.0 (709.5, 2942.3)	1831.0 (1040.0, 3650.0)	0.048
Peak CK-MB (U/L)	66.0 (33.1, 135.8)	121.0 (73.1, 187.8)	≤0.001
Peak cTNI (pg/ml)	2.7 (1.3, 7.1)	5.3 (3.5, 8.0)	≤0.001
NRL	3.9 (2.5, 6.6)	6.3 (3.4, 10.9)	≤0.001
WBC (^*∗*^10^9^)	9.91 ± 3.41	9.93 ± 3.60	0.974
Fibrinogen (g/L)	2.7 (2.2, 3.2)	2.6 (2.1, 3.1)	0.614
LDL-C (mmol/L)	3.1 ± 0.9	2.8 ± 0.7	0.061
Blood uric acid (ummol/L)	353.5 (278.5, 419.8)	354.5 (274.5.0, 412.0)	0.735
Fasting blood sugar (mmol/L)	6.2 (5.0, 7.9)	6.9 (6.0,9.3)	0.008
Lipoprotein *α* (mg/dl)	145.5 (86.3, 256.6)	206.7 (88.4, 285.5)	0.273
CCr (ml/min)	94.8 (69.5, 121.1)	81.5 (67.4, 106.8)	0.090
LVEDV (mm)	99.5 (86.8, 107.3)	100.0 (90.0, 120.0)	0.313
LVESV (mm)	52.0 (41.3, 62.8)	52.0 (43.0, 59.3)	0.609
EF (%)	0.42 (0.38, 0.46)	0.41 (0.38, 0.44)	0.104

Bold values are statistically significant (*P* < 0.05).

**Table 2:** Angiographic procedural characteristics of the study population, mean ± SD or *n* (%).

Variable	Control group (*n* = 192)	CSF/NRF group (*n* = 50)	*P*
Preoperative SBP (mmHg)	131.5 (118.0, 145.8)	116.0 (106.8, 132.0)	≤0.001
Preoperative DBP (mmHg)	79.0 (70.0, 89.0)	72.5 (65.0, 84.3)	≤0.001
Intraoperative hypotension	57 (29.7)	29 (58.0)	≤0.001
Position of myocardial infarction	—	—	0.095
Anterior wall	90 (46.9)	19 (39.5)	—
Lateral wall	18 (9.4)	2 (2.3)	—
Inferior wall	83 (43.2)	29 (60.5)	—
Posterior wall	8 (4.2)	6 (14.0)	—
Right ventricle	18 (9.3)	7 (16.3)	—
Infracted arteries (*n*, %)	—	—	0.119
LAD	85 (50.0)	15 (34.9)	—
LCX	11 (6.5)	1 (2.3)	—
RCA	74 (43.5)	27 (62.8)	—
LM involvement	24 (12.5)	3 (6.0)	0.193
Diffuse lesions (*n*, %)	67 (34.9)	16 (32.0)	0.701
Gensini lesions (*n*, %)	63.5 (43.0,88.0)	62.0 (44.5,92.8)	0.806
Stent length (mm)	29.0 (19.0, 43.0)	39.5 (28.0, 51.0)	0.118
Contrast agent dosage (ml)	130 (110, 160)	135 (127, 160)	0.468

Bold values are statistically significant(*P* < 0.05).

**Table 3:** Multivariate logistic regression analysis for potential predictors of CSF/NRF.

Variable	Univariate analysis		Multivariate analysis	
	OR (95% CI)	*P*	OR (95% CI)	*P*
Age (years)	1.003 (0.994, 1.007)	0.647	—	—
Diabetes mellitus (*n*, %)	1.597 (0.825, 3.095)	0.165	—	—
sST2 (ng/dl)	1.017 (1.010, 1.024)	≤0.001	1.011 (1.003, 1.020)	0.009
Peak NT-proBNP (ng/L)	1.000 (1.000, 1.000)	0.362	—	—
Peak CK-MB (U/L)	1.003 (1.000, 1.006)	0.029	0.997 (0.993, 1.002)	0.233
NLR	1.107 (1.038, 1.179)	0.002	1.085 (1.016, 1.158)	0.015
Fasting blood sugar (mmol/L)	1.091 (0.996, 1.195)	0.061	—	—
SBP (mmHg)	0.960 (0.941, 0.978)	≤0.001	1.886 (0.908, 3.917)	0.089
DBP (mmHg)	0.959 (0.932, 0.986)	0.003	1.004 (0.962, 1.048)	0.841
Intraoperative hypotension	3.271 (1.722, 6.211)	≤0.001	2.107 (1.048, 4.236)	0.036

Bold values are statistically significant (*P* < 0.05).

## Data Availability

The data used to support the results of this study are available from the corresponding author upon request.

## Abbreviations

CSF/NRF: Coronary slow flow/no-reflow
sST2: Soluble growth stimulator gene 2 protein
STEMI: ST-elevation myocardial infarction
PCI: Percutaneous coronary intervention
cTFC: Corrected TIMI frame count.
